# Disease activity and its predictors in early inflammatory arthritis: findings from a national cohort

**DOI:** 10.1093/rheumatology/keab107

**Published:** 2021-02-04

**Authors:** Mark Yates, Joanna M Ledingham, Paul Amlani Hatcher, Maryam Adas, Sasha Hewitt, Sam Bartlett-Pestell, Sanketh Rampes, Sam Norton, James B Galloway

**Affiliations:** 1 Centre for Rheumatic Diseases, King's College London; 2 Department Rheumatology, Queen Alexandra Hospital, Portsmouth, UK; 3 British Society for Rheumatology, NEIAA Patient Panel; 4 Healthcare Quality Improvement Partnership; 5 King's College London, Faculty of Life Sciences and Medicine; 6 Department of Rheumatology, King's College London, UK

**Keywords:** Rheumatoid arthritis, early inflammatory arthritis, disease activity, patient factors, outcome measures, socioeconomic factors

## Abstract

**Objectives:**

We set out to characterize patient factors that predict disease activity during the first year of treatment for early inflammatory arthritis (EIA).

**Methods:**

We used an observational cohort study design, extracting data from a national clinical audit. All NHS organizations providing secondary rheumatology care in England and Wales were eligible to take part, with recruitment from 215/218 (99%) clinical commissioning groups (CCGs)/Health Boards. Participants were >16 years old and newly diagnosed with RA pattern EIA between May 2018 and May 2019. Demographic details collected at baseline included age, gender, ethnicity, work status and postcode, which was converted to an area level measure of socioeconomic position (SEP). Disease activity scores (DAS28) were collected at baseline, three and 12 months follow-up.

**Results:**

A total of 7455 participants were included in analyses. Significant levels of CCG/Health board variation could not be robustly identified from mixed effects modelling. Gender and SEP were predictors of low disease activity at baseline, three and 12 months follow-up. Mapping of margins identified a gradient for SEP, whereby those with higher degrees of deprivation had higher disease activity. Black, Asian and Minority Ethnic patients had lower odds of remission at three months follow-up.

**Conclusion:**

Patient factors (gender, SEP, ethnicity) predict disease activity. The rheumatology community should galvanise to improve access to services for all members of society. More data are required to characterize area level variation in disease activity.

**Rheumatology key messages**

- Rheumatologists report varying remission rates in newly diagnosed RA. Drivers behind this are unclear.
- This analysis of NEIAA data characterises variation in disease activity across England and Wales.
- Social deprivation, BAME ethnicity and female gender are predictors of greater disease activity.

## Introduction

Healthcare research has demonstrated that patient demographic factors such as socioeconomic position (SEP) predict morbidity and mortality. Those who have a lower SEP fare worse, coined ‘the health gap’ by Professor Michael Marmot [1]. The NHS long-term plan published in 2019 sought to address this with increased funding for more deprived areas and encouraging local health systems to develop their own plans to tackle inequality and reduce unwarranted variations in care [2]. Despite local and national initiatives, an updated report by Marmot and colleagues indicated that health inequality continues to rise [3]. Patients with lower SEP are less likely to be referred by their general practitioner to secondary care and are more likely to not attend subsequent secondary care appointments [4]. Optimal management of early RA relies upon prompt diagnosis and regular follow-ups for therapy escalation and monitoring, so it is possible that those with lower SEP achieve worse outcomes as a result; a healthcare inequality driving heterogeneity in remission rates.

The care for patients with early inflammatory arthritis (EIA), of which RA is its commonest form, has evolved hugely over the past 40 years. Until the end of the last century, management strategies for RA included admission to hospital for bedrest and casting of affected joints. Progressive joint destruction leading to disability was almost inevitable, as was increased mortality. The advent of disease modifying, and biological therapy has enabled vastly greater control of disease activity and markedly improved functional and radiological outcomes. Rheumatologists can now offer patients the realistic goal of disease remission, the closest current proxy to a cure [5]. Despite the rapid increase in therapy options and the development of robust management guidelines [6–8], clinicians acknowledge heterogeneity in remission rates among patients.

Here we present a report from The National Early Inflammatory Arthritis Audit (NEIAA). The focus is to characterize how patient factors, such as SEP, predict outcomes. We explore the variation in disease activity over time across England and Wales, accounting for patient characteristics.

## Methods

### Sample

In 2017, the Healthcare Quality Improvement Partnership (HQIP) commissioned the British Society for Rheumatology (BSR) to deliver NEIAA, the latest iteration of the national clinical audit of EIA care. This ambitious project measures care quality and patient outcomes of adults with newly diagnosed RA pattern EIA (clinician defined) across England and Wales. A detailed report focused on provider level performance and variation against process measures [9].

NEIAA commenced in May 2018 and is set to continue until at least 2022. Data are collected from providers of rheumatology services in England and Wales. Patients aged 16 or over, seen with suspected EIA between 8 May 2018 and 7 May 2019 were eligible for inclusion. Data at baseline included patient age, gender, smoking status, ethnicity (observer assigned), work status, symptom duration, referral route and compliance with National Institute for Health and Care Excellence (NICE) guidelines for RA management [8]. Comorbidity burden was captured using the rheumatic diseases comorbidity index (RDCI), a validated measure that gives a weighted score based upon history of chronic lung disease, cardiovascular disease, hypertension, diabetes mellitus, cancer, peptic ulcer disease and depression [10].

Clinical information included tender joint count (TJC), swollen joint count (SJC), patient reported visual analogue score (VAS) of global symptom severity, ESR and/or CRP, RF and anti-citrullinated c-peptide antibody (CCP) status, and baseline treatment strategy.

Patient postcode was linked to the index of multiple deprivation (IMD), an area level composite score of SEP included in the models below as a fixed covariate [11]. A higher IMD reflects greater area level deprivation. General practitioner (GP) post code was linked to Clinical Commissioning Groups (CCGs) in England, and Health Boards in Wales. Further data collection at three and 12 months follow-up was conducted for those eligible for RA treatment, and included TJC/SJC, patient VAS, ESR and/or CRP and treatment choices.

TJC, SJC, ESR and VAS were used to calculate the disease activity score (DAS28), a validated measure of disease activity in RA [12], at baseline, three and 12 months follow-up in patients with RA pattern EIA. Patients were in disease remission if their DAS28 score was <2.6 [13].

All NHS rheumatology departments across England and Wales are required to participate in NEIAA. Full details of the data collection can be found in the project’s annual report [9].

### Statistical analyses

#### Variation in remission rates

Shapefiles detailing coordinates of English CCGs and Welsh Health Boards were downloaded from the Office for National Statistics (ONS) website [14]. The coordinate files were merged using ArcGIS mapping software [15], allowing visualization of both English and Welsh data on the same map. CCG/Health Board mean DAS28 scores and remission rates were mapped at baseline, three and 12 months follow-up using the grmap Stata™ package, an update of the spmap program developed in 2007 [16].

In order to identify statistically significant levels of variation, estimated baseline, three-month and 12-month remission rates were calculated from mixed effects models that apply an empirical Bayes shrinkage estimator [17]. This was performed at two levels: (i) English CCG and Welsh Health Board level; and (ii) Trust level (England only). Using an empirical Bayes shrinkage estimator was appropriate as it allows modelling of performance between groups with variable sample sizes, and also allows inclusion of groups that perfectly predict the outcome of interest.

The baseline model was adjusted for: age; gender; ethnicity; comorbidity; SEP; work status; smoking status; RF/CCP antibody status; referral route; symptom duration; time to referral; and time to rheumatology review. The three-month and 12-month models were adjusted for: age; gender; ethnicity; comorbidity; SEP; work status; smoking status; RF/CCP antibody status; referral route; time to referral; time to treatment; symptom duration; corticosteroid use; and baseline disease modifying therapy (DMARD) strategy. Baseline DMARD strategy was categorized as none, single therapy or combination therapy. An interaction term for corticosteroid use and DMARD strategy was included.

A CCG/Health Board or Trust was considered to have a high rate of remission if their predicted 95% CI lower bound was greater than the overall mean. Similarly, they were considered to have a low rate of remission if their predicted 95% CI upper bound was less than the overall mean. Findings were plotted on caterpillar graphs.

In line with ONS small numbers reporting guidance, CCGs, Health Boards or Trusts with five or fewer patients recruited to NEIAA were excluded from mapping and caterpillar graphs to ensure patient confidentiality was maintained [18].

#### Predictors of remission

Mixed effects models used to characterize variation were examined to determine factors that associated with DAS28 at baseline, three months and 12 months, with CCG/Health Board included as a random effect. Factors found to be predictive were further explored and visualized by calculating and plotting predictive margins.

Two sensitivity analyses were conducted: (i) the models were rerun as mixed effects linear regression models with DAS28 score at baseline, three months and 12 months as outcome variables; and (ii) remission models were rerun following multiple imputation to account for missing predictor and outcome data. The following variables were used to perform the multiple imputation: age, gender, Trust/Health Board, region, smoking status, and ethnicity. Missing DAS28 data were imputed using linear regression.

### Patient and public involvement

A patient panel was consulted regularly during the design phase of NEIAA. Close liaison has continued since data collection began. The Chair of the patient panel is one of the authors of this manuscript. He offered the patient’s perspective on the analyses performed, and the manuscript write-up.

## Results

### Cohort details

In year one of NEIAA, a total of 21 148 patients with suspected EIA were recruited. Patients were recruited from 209/211 CCGs in England, and from all seven Health Boards in Wales. A total of 7455/21 148 (35%) from 215 CCGs/Health Boards were commenced on a treatment pathway for RA. The mean age of the cohort at recruitment was 56.7 years [(s.d.) 16.1], 62.3% were female and 86.8% were white ethnicity. 56.3% were either RF or CCP antibody positive. Mean DAS28 scores reduced over follow up, from 4.7 (s.d. 1.5) at baseline, to 2.8 (s.d. 1.3) at 12 months. A total of 5861/7455 (78.6%) patients were commenced on DMARD therapy, of whom 3085/5861 (52.6%) received treatment within 6 weeks of referral. In total, 4279/7198 (59.4%) were commenced on therapy at baseline appointment, with monotherapy used more than combination therapy (83% *vs* 17%). Further cohort details can be found in Table 1. There were substantial levels of missing DAS28 data, with 10.1% missing at baseline, 34.9% at three months and 68% at 12 months.

**Table 1:** Demographic and clinical characteristics of patients with RA pattern EIA

	Value	*n* missing (%)
*n* = 7455		
Age, mean (s.d.)	56.7 (16.1)	0
Female (%)	4647 (62.3%)	0
IMD decile, mean (s.d.)	5.4 (2.9)	719 (9.6%)
BAME (%)	987 (13.2%)	0
Paid work >20 h/week (%)	3529 (48.0%)	104 (1.4%)
Current smoker (%)	1422 (19.1%)	0
≥1 comorbidity (%)	3000 (40.2%)	100 (1.3%)
Seropositive (%)	3779 (56.3%)	742 (10.0%)
Symptom duration (%)		68 (0.9%)
<1 month	637 (8.5%)	
1–6 months	4055 (54.4%)	
>6 months	2695 (36.2%)	
Baseline DAS28, mean (s.d.)	4.7 (1.5)	677 (10.1%)
3 months DAS28, mean (s.d.)	3.3 (1.5)	2,603 (34.9%)
12 months DAS28, mean (s.d.) (*n* = 4273)	2.8 (1.3)	2902 (68.0%)
Primary care referral within 3 days (%)	2908 (39%)	107 (1.4%)
Rheumatology review within 21 days of referral (%)	2977 (40%)	51 (0.7%)
Commence therapy within 42 days of referral (%)	3085 (41.4%)	1746 (23.4%)
Baseline disease modifying therapy (%)		257 (3.5%)
No therapy	2919 (39.1%)	
Monotherapy	3556 (47.7%)	
Combination therapy	723 (9.7%)	
Baseline corticosteroids (%)	5151 (69.1%)	206 (2.8%)

Patients were considered seropositive if they had either RF or anti-citrullinated c-peptide antibodies.

BAME: black, Asian, and minority ethnic; DAS28: disease activity score; IMD: index of multiple deprivation.

### Variation in disease activity over time

**Fig. 1:**
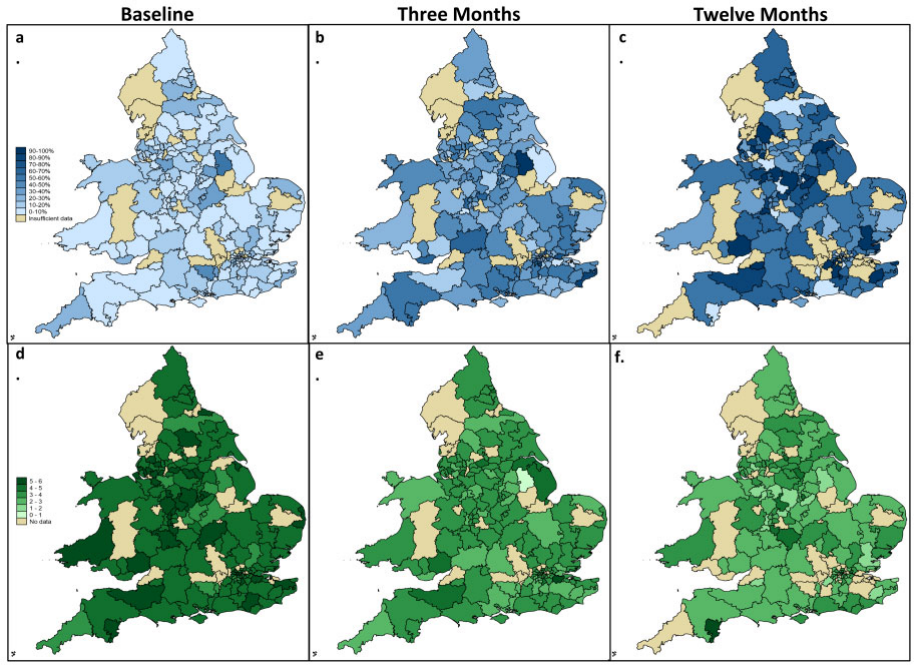
CCG/Health Board remission rates and mean DAS28 scores CCG/Health Board remission rates mapped at **(a)** baseline, **(b)** three months and **(c)** 12 months. Darker colours represent higher remission rates. CCG/Health Board mean DAS28 scores at **(d)** baseline, **(e)** three months and **(f)** 12 months. Darker colours represent higher disease activity. Khaki represents CCGs/Health Boards with <6 patients. CCG: clinical commissioning group; DAS28: disease activity score.

Before considering any impact of patient factors, we analysed unadjusted CCG/Health Board remission rates. These varied widely across England and Wales, with remission rates increasing at 3 and 12 months. Fig. 1 details mapping of observed CCG/Health Board remission and mean DAS28 scores. At baseline, 10.6% had a DAS28 score of <2.6. The range across CCG/Health Boards was 0% to 57.1% (s.d. 9.3). At three-month follow-up, 36.2% were in remission (range 0% to 100%, s.d. 16), rising to 53.6% (range 0% to 100%, s.d. 28.1) at 12 months.

We then modelled variation accounting for patient characteristics. The results are displayed in caterpillar graphs. These show the observed and modelled empirical Bayes estimates per CCG/Health Board and Trust and are included in the Supplementary Material, available at *Rheumatology* online.

The striking differences observed in disease activity across the country in unadjusted data are no longer clinically significant in the adjusted models.

### Predictors of disease activity

The second step in our analysis was to consider patient characteristics and how these associated with disease activity over time.

#### Model 1: baseline disease severity

At baseline, younger age, male gender, fewer comorbidities, paid employment and lower deprivation associated with milder disease at presentation. Patients seen by a rheumatologist within three weeks of referral had lower odds of remission at baseline. The associations were preserved following multiple imputation. Full details of the model with odds ratios are in Table 2.

**Table 2:** Mixed effects model identifying predictors of baseline remission^a^

Baseline remission	Odds ratio	*P*-value	95% CI
Age	0.98	<0.0001	0.97, 0.99
Female gender	0.67	<0.0001	0.55, 0.82
BAME	0.95	0.7	0.70, 1.29
Smoking (ref: current smoker)			
Ex-smoker	0.88	0.4	0.66, 1.17
Never smoked	0.89	0.4	0.69, 1.16
Paid work	1.42	0.002	1.13, 1.78
IMD	1.08	<0.0001	1.04, 1.13
Comorbidity	0.83	0.002	0.74, 0.94
Seropositive	0.98	0.9	0.81, 1.20
Symptom duration (ref: <1 month)			
1, 6 months	0.60	0.002	0.43, 0.83
>6 months	0.72	0.06	0.51, 1.01
Referred via EIA pathway	0.90	0.3	0.73, 1.11
Prompt referral	0.90	0.3	0.73, 1.11
Prompt specialist review	0.75	0.008	0.61, 0.93

a Baseline remission implies a DAS28 at diagnosis <2.6. Lower age, male gender, paid work, high SEP, fewer comorbidities, and prompt rheumatology review all associated with baseline remission. Symptom duration also appeared to associate with remission, but the relationship was non-linear. Prompt referral was defined as a referral that was sent within three days of primary care review. Prompt review was defined as a rheumatology review within 21 days of primary care referral receipt. BAME: black, Asian, and minority ethnic; IMD: index of multiple deprivation.

#### Model 2: three months remission

At three-month follow-up, male gender, white British ethnicity, lower deprivation, and seropositive autoantibodies associated with disease remission. Patients who were commenced on therapy promptly were more likely to achieve remission. Following imputation, seropositivity lost its association, whereas fewer comorbidities and baseline corticosteroids crossed the 0.05 threshold for statistical significance. Full details can be found in Table 3.

**Table 3:** Mixed effects model identifying predictors of three months remission

3 months remission	Odds ratio	*P*-value	95% CI
Age	1.00	0.9	0.99, 1.01
Female gender	0.65	<0.0001	0.55, 0.76
BAME	0.73	0.010	0.57, 0.93
Smoking (ref: current smoker)			
Ex-smoker	1.16	0.2	0.93, 1.43
Never smoked	1.08	0.5	0.88, 1.33
Paid work	1.05	0.6	0.88, 1.25
IMD	1.06	<0.0001	1.04, 1.09
Comorbidity	0.96	0.3	0.89, 1.03
Seropositive	1.33	<0.0001	1.14, 1.56
Symptom duration (ref: <1 month)			
1–6 months	1.13	0.4	0.87, 1.48
>6 months	0.91	0.5	0.68, 1.21
Prompt referral	1.09	0.3	0.94, 1.27
Prompt therapy commencement	1.27	0.003	1.08, 1.49
Initial DMARD regimen (ref: no DMARD)			
Monotherapy	1.34	0.1	0.94, 1.90
Combination therapy	1.04	0.9	0.48, 2.25
Baseline corticosteroids	0.99	0.9	0.72, 1.35
DMARD monotherapy + corticosteroids	0.80	0.3	0.54, 1.19
DMARD combination therapy + corticosteroids	1.07	0.9	0.47, 2.41

Male gender, BAME, high SEP, seropositivity and prompt therapy commencement all associated with three months remission. Prompt therapy commencement was defined as commencing therapy within 42 days of referral. BAME: black, Asian, and minority ethnic; DMARD: disease modifying anti-rheumatic drug; IMD: index of multiple deprivation.

#### Model 3: 12 months remission

At 12-month follow-up, male gender, fewer comorbidities, lower deprivation, and being an ex-smoker (compared with a current smoker) predicted disease remission. In the imputed model, these effects were preserved. Full details are in Table 4.

**Table 4:** Mixed effects model identifying predictors of 12 months remission

12 months remission	Odds ratio	*P*-value	95% CI
Age	1.01	0.3	0.99, 1.02
Female gender	0.61	0.001	0.46, 0.81
BAME	0.88	0.5	0.58, 1.32
Smoking (ref: current smoker)			
Ex-smoker	1.59	0.02	1.08, 2.32
Never smoked	1.38	0.09	0.95, 1.99
Paid work	1.01	0.7	0.79, 1.44
IMD	1.06	0.02	1.01, 1.11
Comorbidity	0.83	0.005	0.73, 0.94
Seropositive	1.12	0.4	0.85, 1.48
Symptom duration (ref: <1 month)			
1–6 months	1.10	0.7	0.70, 1.74
>6 months	0.75	0.3	0.46, 1.23
Prompt referral	1.10	0.5	0.84, 1.44
Prompt therapy commencement	1.05	0.7	0.80, 1.38
Initial DMARD regimen (ref: no DMARD)			
Monotherapy	1.41	0.3	0.75, 2.65
Combination therapy	1.61	0.5	0.41, 6.25
Baseline corticosteroids	0.82	0.5	0.47, 1.43
DMARD monotherapy + corticosteroids	0.88	0.7	0.44, 1.78
DMARD combination therapy + corticosteroids	0.88	0.9	0.21, 3.61

Male gender, smoking status, SEP and comorbidity all associated with 12 months remission. The relationship with smoking status was non-linear. BAME: black, Asian, and minority ethnic; DMARD: disease modifying anti-rheumatic drug; IMD: index of multiple deprivation.

**Fig. 2:**
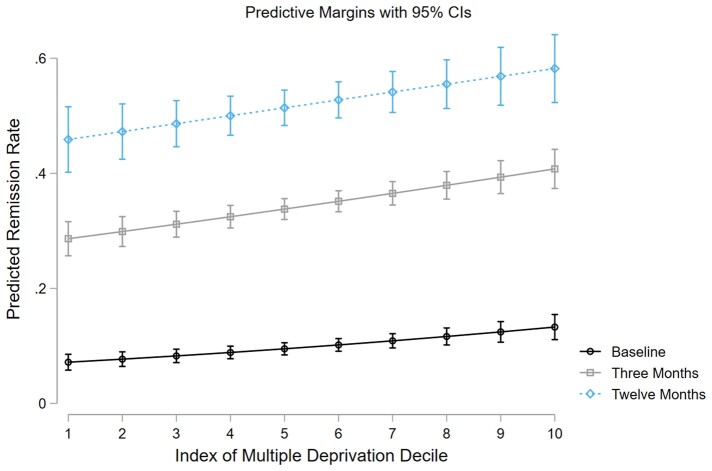
Predicted margins of IMD at baseline, three-month and 12-month follow-up Plotted margins of IMD from the three logistic regression models, predicting remission at baseline (black line), three months (grey line) and 12 months (blue dashed line) with vertical lines representing 95% CIs. All three models indicate that the likelihood of remission increases as IMD decile increases, which corresponds to reducing deprivation.

The predictive margins of IMD deciles are plotted for baseline, three months and 12 months remission in Fig. 2.

Mixed effects linear regression modelling was performed and identified the same factors that predicted remission also predicted mean DAS28, with the addition of comorbidity and corticosteroids that predicted DAS28 at three months. Further details and imputed results can be found in the Supplementary Material, available at *Rheumatology* online.

Further sensitivity analyses were conducted to assess if missing DAS28 data predicted IMD. There was no association at baseline, three or 12 months.

Demographic and clinical characteristics stratified by missing DAS28 at baseline, three months and 12 months are detailed in the Supplementary Material, available at *Rheumatology* online. Gender and IMD were similar in those with complete or incomplete data. Comorbidity burden was lower in those with a missing baseline DAS28, but there were no differences at three and 12 months. A higher proportion of participants with missing DAS28 at all time points identified as black, Asian, and minority ethnic (BAME).

## Discussion

This work presents a contemporary view of variation in and predictors of remission rates in newly diagnosed rheumatoid pattern EIA from NEIAA, a national prospective cohort. Modelling of estimated remission rates highlighted that the variation observed between CCGs/Health Boards was not statistically significant once we had accounted for patient characteristics, including age, gender, SEP and ethnicity. A caveat to the interpretation is that there were high levels of incomplete DAS28 data, particularly at three months and 12 months which may be obscuring underlying variation between CCGs/Health Boards. Future analyses with data from year two of NEIAA will assist in clarifying this.

The findings confirm the hypothesis that patient level factors are predictive of achieving remission. Male gender was a strong predictor at baseline, three months and 12 months in adjusted mixed effects modelling. It has been previously reported that women have worse disease at diagnosis, and lower rates of remission over time [19–21], but the extent of the effect reported here is striking, with men at least 30% more likely to be in remission at all time points. RA affects women more often than men and animal models suggest that oestrogen may cause T-cell activation [22], which may be an explanatory factor, although other possible factors such as unconscious bias may also play a role. The discrepancy between genders becomes more pronounced with longer follow-up, which suggests women may be less responsive to initial therapy than men. This is supported by a meta-analysis of conventional disease-modifying therapy phase III randomized controlled trials (RCTs), which found that women had a lower treatment response than men [23].

SEP associated with remission at all time points in our analyses, with those living in more deprived areas less likely to achieve remission. This is portrayed in the margins plot, which highlights a social gradient in NHS rheumatology care, where those who are more deprived experience worse outcomes. It is well established that more deprived individuals experience worse health outcomes [3], and reducing this source of health inequality is a key focus of the NHS long-term plan [2].

Ethnicity associated with remission at three months, which is consistent with findings from observational studies in the United States where non-white participants were less likely to achieve remission [24]. There is under-representation of ethnic minorities in RCTs, and the evidence base for therapies may not be valid for some ethnic groups [25]. It is crucial that RCT sample demographics reflect target populations so there can be confidence in the external validity of the findings to clinical practice in areas serving heterogeneous populations.

Comorbidity burden measured by the RDCI associated with low disease activity at baseline and remission at 12 months follow up. This finding is consistent with previous cohort studies [26]. This may be due to fewer treatment options being available to patients with a higher comorbidity burden. Some comorbidities will be modifiable, offering a chance to improve the likelihood of achieving remission. This underlines the importance of patients with early RA receiving care not solely focused on their articular symptoms.

Trial data indicate that achieving the best possible clinical outcomes in early RA relies on multiple factors including prompt diagnosis, early treatment commencement and regular attendance for therapy monitoring and escalation [27]. A key concept of patient engagement with care is candidacy. Candidacy refers to how individuals and care providers perceive eligibility for healthcare services [28]. Permeability, a construct within candidacy, relates to the ease of access to a health service. Permeability is higher if lower education or resources (such as a car) are required to enable access [4]. The often-nonspecific nature of symptoms in early RA and the need for regular follow-up appointments require a relatively high degree of candidacy to achieve the best outcomes.

The role of patient factors in candidacy is complex. Gender and its impact on outcomes and healthcare utilization has been extensively studied, with variation depending on the disease area [29]. Ethnicity also appears to impact on candidacy, with ethnic minorities having lower access to care [30]. This is likely driven by lower rates of native language fluency [31], and higher levels of deprivation in ethnic minority groups [32].

Individuals who are more deprived have impaired candidacy due to four key reasons [4]:

- Financial implications of attending appointments. This can be due to direct transport costs as well as reduced pay from attending appointments. Those with lower SEP are more likely to work in hourly rate occupations with little or no sick pay benefits.
- Patient knowledge and literacy may mean individuals are less aware of services that may be of benefit and might also be less able to explain their symptoms.
- Clinician perception of benefit. More deprived individuals are disproportionately impacted by negative lifestyle factors such as smoking, alcohol intake and physical activity [33]. Clinicians are less likely to offer treatment to patients with these risk factors. Previous work using national clinical audit data identified marked variations in early RA prescribing [34], which may in part be driven by (often justified) clinician concerns regarding risk factors that disproportionately impact those with lower SEP.
- Patient alienation. Patients may feel they have no commonality with their clinician, so feel uncomfortable sharing information with them.

To address the impact of individual factors in EIA care, policy makers, commissioners and clinicians should focus on: delivering flexible appointment times with evening and weekend availability; patient-friendly public awareness campaigns of the early signs and symptoms of EIA; promotion and adherence to evidence based therapy guidelines; and encourage medical schools to seek applications from individuals drawn from all societal groups so the medical workforce better reflects the population it serves. The expansion of first contact practitioners in musculoskeletal care offers patients an alternative route to rheumatology secondary care referral that may assist in improving service permeability [35].

This study benefits from a large sample size drawn from almost all rheumatology departments across England and Wales. The models presented are extensively adjusted, accounting for patient level factors and hierarchies within the data, giving the authors confidence in the robustness of the findings. A limitation is the degree of incomplete data, particularly at 12-month follow-up. While findings were consistent following multiple imputation, it is possible that the data were missing not at random. Notably, ethnicity was different in those with complete and incomplete DAS28 data, which may have contributed to the association between BAME and DAS28. There were no significant differences in gender and deprivation, suggesting the primary findings presented here are robust. The degree of missing data is substantial and prevented conclusions to be drawn on CCG level variation in 12-month remission. As NEIAA approaches the end of year 2, data quality and quantity will improve, allowing us to further characterize care variation.

We measured SEP using the IMD. This is an area level surrogate for individual SEP, so is at risk of an ecological bias where area level data is used to make inferences about individuals within that area. In RA, however, IMD is advantageous as the female predominance of the disease means individual measures such as occupation and income are more likely to be biased.

This paper identifies that patient-level factors including gender, SEP and ethnicity predict remission. There will be a degree of variation in disease activity based on genetic and environmental differences that cannot be altered. Despite this, it is incumbent on commissioners and clinicians to promote equality of access to services by taking patient demographics into account.

## Supplementary Material

keab107_supplementary_data
