# Supplementary material for: Disease activity and its predictors in early inflammatory arthritis: findings from a national cohort
Source: Rheumatology (Oxford). 2021 Feb 4;60(10):4811–20. doi: 10.1093/rheumatology/keab107 (PMC8487309; doi:10.1093/rheumatology/keab107)
Supplement: keab107_supplementary_data [file keab107_supplementary_data.docx]

Supplementary Material

12 Months

3 Months

3 Months

Baseline

Baseline

Caterpillar plots of CCG/Health board estimated remission rates at baseline and three months. Caterpillar plots of Trust estimated remission rates at baseline, three months, and 12 months follow up. Black horizontal line =sample mean, grey markers = observed remission rates, blue markers = estimated remission rates from model, red lines = 95% confidence interval of estimated remission rates. CCG = clinical commissioning group.

Insufficient data

Figure 1. Caterpillar graphs of observed and modelled remission.

*Table 1. Demographics and clinical characteristics stratified by missing DAS28 at baseline*

|  | **Baseline DAS28 complete** | **Baseline DAS28 missing** | **p-value** | **Test** |
| --- | --- | --- | --- | --- |
|  | N=6,778 | N=677 |  |  |
| **Age** | 57 (16) | 54 (16) | <0.001 | Two sample t test |
| **Male gender** | 2,539 (37.5%) | 269 (39.7%) | 0.24 | Pearson's chi-squared |
| **BAME** | 867 (13%) | 120 (18%) | <0.001 | Pearson's chi-squared |
| **Smoking status** |  |  | <0.001 | Pearson's chi-squared |
| **Current smoker** | 1,317 (19%) | 105 (16%) |  |  |
| **Ex-smoker** | 1,933 (29%) | 181 (27%) |  |  |
| **Never smoked** | 3,072 (45%) | 263 (39%) |  |  |
| **Not known** | 456 ( 7%) | 128 (19%) |  |  |
| **Paid work >20h/week** | 3,199 (47%) | 330 (55%) | <0.001 | Pearson's chi-squared |
| **IMD Decile** |  |  | 0.054 | Pearson's chi-squared |
| **1** | 633 (10%) | 75 (12%) |  |  |
| **2** | 638 (10%) | 83 (14%) |  |  |
| **3** | 608 (10%) | 48 ( 8%) |  |  |
| **4** | 583 (10%) | 51 ( 8%) |  |  |
| **5** | 615 (10%) | 58 (10%) |  |  |
| **6** | 606 (10%) | 57 ( 9%) |  |  |
| **7** | 630 (10%) | 70 (12%) |  |  |
| **8** | 617 (10%) | 45 ( 7%) |  |  |
| **9** | 660 (11%) | 58 (10%) |  |  |
| **10** | 545 ( 9%) | 56 ( 9%) |  |  |
| **RDCI** |  |  | <0.001 | Pearson's chi-squared |
| **0** | 3,945 (58%) | 410 (71%) |  |  |
| **1** | 1,451 (21%) | 100 (17%) |  |  |
| **2** | 849 (13%) | 43 ( 7%) |  |  |
| **3** | 348 ( 5%) | 17 ( 3%) |  |  |
| **4** | 128 ( 2%) | 6 ( 1%) |  |  |
| **5** | 45 ( 1%) | 0 ( 0%) |  |  |
| **6** | 12 ( 0%) | 0 ( 0%) |  |  |
| **7** | 0 ( 0%) | 1 ( 0%) |  |  |
| **Seropositive** | 3,611 (57%) | 168 (44%) | <0.001 | Pearson's chi-squared |
| **Symptom Duration** |  |  | <0.001 | Pearson's chi-squared |
| **<1 month** | 606 ( 9%) | 31 ( 5%) |  |  |
| **<6 month** | 3,756 (56%) | 299 (48%) |  |  |
| **>6 month** | 2,402 (36%) | 293 (47%) |  |  |
| **Referral via EIA pathway** | 4,055 (60%) | 372 (58%) | 0.26 | Pearson's chi-squared |
| **Primary care referral within 3 days** | 2,665 (40%) | 243 (40%) | 0.86 | Pearson's chi-squared |
| **Rheumatology review within 21 days of referral** | 2,769 (41%) | 208 (32%) | <0.001 | Pearson's chi-squared |

*Table 2. Demographics and clinical characteristics stratified by missing DAS28 at 3 months.*

|  | **3 months DAS28 complete** | **3 months DAS28 missing** | **p-value** | **Test** |
| --- | --- | --- | --- | --- |
|  | N=4,852 | N=2,603 |  |  |
| **Age** | 58 (16) | 55 (17) | <0.001 | Two sample t test |
| **Male gender** | 1,807 (37.2%) | 1,001 (38.5%) | 0.30 | Pearson's chi-squared |
| **BAME** | 579 (12%) | 408 (16%) | <0.001 | Pearson's chi-squared |
| **Smoking status** |  |  | <0.001 | Pearson's chi-squared |
| **Current smoker** | 947 (20%) | 475 (18%) |  |  |
| **Ex-smoker** | 1,427 (29%) | 687 (26%) |  |  |
| **Never smoked** | 2,162 (45%) | 1,173 (45%) |  |  |
| **Not known** | 316 ( 7%) | 268 (10%) |  |  |
| **Paid work >20h/week** | 2,257 (47%) | 1,272 (50%) | 0.006 | Pearson's chi-squared |
| **IMD Decile** |  |  | 0.056 | Pearson's chi-squared |
| **1** | 442 (10%) | 266 (12%) |  |  |
| **2** | 449 (10%) | 272 (12%) |  |  |
| **3** | 445 (10%) | 211 ( 9%) |  |  |
| **4** | 437 (10%) | 197 ( 9%) |  |  |
| **5** | 451 (10%) | 222 (10%) |  |  |
| **6** | 448 (10%) | 215 ( 9%) |  |  |
| **7** | 458 (10%) | 242 (11%) |  |  |
| **8** | 452 (10%) | 210 ( 9%) |  |  |
| **9** | 482 (11%) | 236 (10%) |  |  |
| **10** | 382 ( 9%) | 219 (10%) |  |  |
| **RDCI** |  |  | 0.12 | Pearson's chi-squared |
| **0** | 2,822 (58%) | 1,533 (61%) |  |  |
| **1** | 1,020 (21%) | 531 (21%) |  |  |
| **2** | 619 (13%) | 273 (11%) |  |  |
| **3** | 248 ( 5%) | 117 ( 5%) |  |  |
| **4** | 93 ( 2%) | 41 ( 2%) |  |  |
| **5** | 32 ( 1%) | 13 ( 1%) |  |  |
| **6** | 9 ( 0%) | 3 ( 0%) |  |  |
| **7** | 0 ( 0%) | 1 ( 0%) |  |  |
| **Seropositive** | 2,744 (60%) | 1,035 (48%) | <0.001 | Pearson's chi-squared |
| **Symptom Duration** |  |  | <0.001 | Pearson's chi-squared |
| **<1 month** | 423 ( 9%) | 214 ( 8%) |  |  |
| **<6 month** | 2,745 (57%) | 1,310 (51%) |  |  |
| **>6 month** | 1,670 (35%) | 1,025 (40%) |  |  |
| **Referral via EIA pathway** | 2,920 (60%) | 1,507 (59%) | 0.19 | Pearson's chi-squared |
| **Primary care referral within 3 days** | 1,904 (39%) | 1,004 (40%) | 0.81 | Pearson's chi-squared |
| **Rheumatology review within 21 days of referral** | 2,052 (42%) | 925 (36%) | <0.001 | Pearson's chi-squared |

*Table 3. Demographics and clinical characteristics stratified by missing DAS28 at 12 months.*

|  | **12 months DAS28 complete** | **12 months DAS28 missing** | **p-value** | **Test** |
| --- | --- | --- | --- | --- |
|  | N=1,371 | N=2,902 |  |  |
| **Age** | 58 (15) | 56 (17) | <0.001 | Two sample t test |
| **Male gender** | 500 (36.5%) | 1,089 (37.5%) | 0.50 | Pearson's chi-squared |
| **BAME** | 156 (11%) | 418 (14%) | 0.007 | Pearson's chi-squared |
| **Smoking status** |  |  | 0.045 | Pearson's chi-squared |
| **Current smoker** | 280 (20%) | 550 (19%) |  |  |
| **Ex-smoker** | 415 (30%) | 803 (28%) |  |  |
| **Never smoked** | 589 (43%) | 1,315 (45%) |  |  |
| **Not known** | 87 ( 6%) | 234 ( 8%) |  |  |
| **Paid work >20h/week** | 625 (46%) | 1,355 (48%) | 0.25 | Pearson's chi-squared |
| **IMD Decile** |  |  | 0.92 | Pearson's chi-squared |
| **1** | 141 (11%) | 299 (11%) |  |  |
| **2** | 138 (11%) | 313 (12%) |  |  |
| **3** | 124 (10%) | 240 ( 9%) |  |  |
| **4** | 127 (10%) | 245 ( 9%) |  |  |
| **5** | 123 (10%) | 264 (10%) |  |  |
| **6** | 121 ( 9%) | 270 (10%) |  |  |
| **7** | 127 (10%) | 266 (10%) |  |  |
| **8** | 118 ( 9%) | 260 (10%) |  |  |
| **9** | 155 (12%) | 301 (11%) |  |  |
| **10** | 104 ( 8%) | 246 ( 9%) |  |  |
| **RDCI** |  |  | 0.91 | Pearson's chi-squared |
| **0** | 800 (59%) | 1,698 (59%) |  |  |
| **1** | 295 (22%) | 604 (21%) |  |  |
| **2** | 158 (12%) | 348 (12%) |  |  |
| **3** | 80 ( 6%) | 142 ( 5%) |  |  |
| **4** | 23 ( 2%) | 43 ( 2%) |  |  |
| **5** | 8 ( 1%) | 20 ( 1%) |  |  |
| **6** | 2 ( 0%) | 4 ( 0%) |  |  |
| **7** | 0 ( 0%) | 1 ( 0%) |  |  |
| **Seropositive** | 811 (63%) | 1,370 (53%) | <0.001 | Pearson's chi-squared |
| **Symptom Duration** |  |  | 0.023 | Pearson's chi-squared |
| **<1 month** | 121 ( 9%) | 240 ( 8%) |  |  |
| **<6 month** | 797 (58%) | 1,551 (54%) |  |  |
| **>6 month** | 452 (33%) | 1,066 (37%) |  |  |
| **Referral via EIA pathway** | 815 (59%) | 1,668 (58%) | 0.46 | Pearson's chi-squared |
| **Primary care referral within 3 days** | 505 (37%) | 1,092 (38%) | 0.38 | Pearson's chi-squared |
| **Rheumatology review within 21 days of referral** | 544 (40%) | 1,076 (37%) | 0.11 | Pearson's chi-squared |

**Remission models with imputed data**

Incomplete covariates and DAS28 data were estimated via multiple imputation with chained equations using age, gender, ethnicity, smoking, Trust and region as predictor variables. Twenty iterations were performed with results combined following Rubin’s rules (1).

Table 4.Predictors of baseline remission using imputed data.

| Baseline remission | Odds Ratio | p value | 95% Confidence interval |
| --- | --- | --- | --- |
| Age | 0.98 | <0.0001 | 0.97 to 0.98 |
| Female gender | 0.67 | <0.0001 | 0.56 to 0.80 |
| BAME | 1.01 | 0.9 | 0.78 to 1.30 |
| Smoking (ref: current smoker) | | | |
| Ex-smoker | 1.05 | 0.7 | 0.82 to 1.34 |
| Never smoked | 1.00 | 0.9 | 0.79 to 1.25 |
| Paid work | 1.38 | 0.001 | 1.15 to 1.66 |
| IMD | 1.09 | <0.0001 | 1.05 to 1.13 |
| Comorbidity | 0.83 | <0.0001 | 0.74 to 0.91 |
| Seropositive | 0.95 | 0.5 | 0.80 to 1.12 |
| Symptom duration (ref: <1 month) | |  |  |
| 1 to 6 months | 0.75 | 0.05 | 0.56 to 1.00 |
| >6 months | 0.91 | 0.6 | 0.67 to 1.23 |
| Referred via EIA pathway | 0.90 | 0.2 | 0.75 to 1.07 |
| Prompt referral | 0.99 | 0.9 | 0.84 to 1.18 |
| Prompt review | 0.77 | 0.003 | 0.64 to 0.91 |

Table 5. Predictors of three months remission using imputed data.

| Three months remission | Odds Ratio | p value | 95% Confidence interval |
| --- | --- | --- | --- |
| Age | 1.00 | 0.9 | 0.99 to 1.00 |
| Female gender | 0.66 | <0.0001 | 0.58 to 0.74 |
| BAME | 0.79 | 0.02 | 0.64 to 0.96 |
| Smoking (ref: current smoker) | | | |
| Ex-smoker | 1.15 | 0.1 | 0.97 to 1.36 |
| Never smoked | 1.16 | 0.08 | 0.98 to 1.37 |
| Paid work | 1.09 | 0.2 | 0.96 to 1.25 |
| IMD | 1.06 | <0.0001 | 1.04 to 1.09 |
| Comorbidity | 0.93 | 0.04 | 0.87 to 1.00 |
| Seropositive | 1.12 | 0.09 | 0.98 to 1.28 |
| Symptom duration (ref: <1 month) | | | |
| 1 to 6 months | 0.97 | 0.8 | 0.80 to 1.18 |
| >6 months | 0.82 | 0.06 | 0.67 to 1.01 |
| Prompt referral | 1.02 | 0.7 | 0.91 to 1.15 |
| Prompt therapy commencement | 1.20 | 0.005 | 1.06 to 1.37 |
| Initial DMARD regimen (ref: no DMARD) | | | |
| Monotherapy | 0.84 | 0.1 | 0.67 to 1.05 |
| Combination therapy | 0.72 | 0.2 | 0.41 to 1.26 |
| Baseline Corticosteroids | 0.74 | 0.001 | 0.61 to 0.89 |
| DMARD monotherapy + corticosteroids | 1.02 | 0.9 | 0.78 to 1.33 |
| DMARD combination therapy + corticosteroids | 1.42 | 0.3 | 0.78 to 2.58 |

Table 6. Predictors of 12 months remission using imputed data.

| 12 months remission | Odds Ratio | p value | 95% Confidence interval |  |
| --- | --- | --- | --- | --- |
| Age | 1.01 | 0.3 | 0.99 to 1.02 |  |
| Female gender | 0.61 | 0.001 | 0.46 to 0.81 |  |
| BAME | 0.88 | 0.5 | 0.58 to 1.32 |  |
| Smoking (ref: current smoker) | | | | |
| Ex-smoker | 1.59 | 0.02 | 1.08 to 2.32 |  |
| Never smoked | 1.38 | 0.09 | 0.95 to 1.99 |  |
| Paid work | 1.01 | 0.7 | 0.79 to 1.44 |  |
| IMD | 1.06 | 0.02 | 1.01 to 1.11 |  |
| Comorbidity | 0.83 | 0.005 | 0.73 to 0.94 |  |
| Seropositive | 1.12 | 0.4 | 0.85 to 1.48 |  |
| Symptom duration (ref: <1 month) | | | |  |
| 1 to 6 months | 1.10 | 0.7 | 0.70 to 1.74 |  |
| >6 months | 0.75 | 0.3 | 0.46 to 1.23 |  |
| Prompt referral | 1.10 | 0.5 | 0.84 to 1.44 |  |
| Prompt therapy commencement | 1.05 | 0.7 | 0.80 to 1.38 |  |
| Initial DMARD regimen (ref: no DMARD) | | | |  |
| Monotherapy | 1.41 | 0.3 | 0.75 to 2.65 |  |
| Combination therapy | 1.61 | 0.5 | 0.41 to 6.25 |  |
| Baseline Corticosteroids | 0.82 | 0.5 | 0.47 to 1.43 |  |
| DMARD monotherapy + corticosteroids | 0.88 | 0.7 | 0.44 to 1.78 |  |
| DMARD combination therapy + corticosteroids | 0.88 | 0.9 | 0.21 to 3.61 |  |

**Mixed effects linear regression models to identify predictors of DAS28**

Table 7. Predictors of baseline DAS28.

| Baseline DAS28 | Beta-coefficient | p value | 95% Confidence interval |
| --- | --- | --- | --- |
| Age | 0.16 | <0.0001 | 0.013 to 0.019 |
| Female gender | 0.17 | <0.0001 | 0.082 to 0.25 |
| BAME | 0.025 | 0.7 | -0.11 to 0.16 |
| Smoking (ref: current smoker) | | | |
| Ex-smoker | -0.011 | 0.9 | -0.13 to 0.11 |
| Never smoked | 0.010 | 0.9 | -0.11 to 0.11 |
| Paid work | -0.23 | <0.0001 | -0.33 to -0.14 |
| IMD | -0.056 | <0.0001 | -0.33 to -0.14 |
| Comorbidity | 0.085 | <0.0001 | 0.044 to 0.13 |
| Seropositive | 0.009 | 0.8 | -0.073 to 0.092 |
| Symptom duration (ref: <1 month) | |  |  |
| 1 to 6 months | 0.11 | 0.1 | -0.032 to 0.26 |
| >6 months | -0.11 | 0.2 | -0.27 to 0.042 |
| Referred via EIA pathway | -0.078 | 0.9 | -0.099 to 0.083 |
| Prompt referral | -0.010 | 0.8 | 0.097 to 0.083 |
| Prompt review | 0.23 | <0.0001 | 0.14 to 0.31 |

Table 8. Predictors of three months DAS28.

| Three months DAS28 | Beta-coefficient | p value | 95% Confidence interval |  |
| --- | --- | --- | --- | --- |
| Age | -0.02 | 0.3 | -0.060 to -.0019 |  |
| Female gender | 0.29 | <0.0001 | 0.18 to 0.39 |  |
| BAME | 0.24 | 0.002 | 0.087 to 0.39 |  |
| Smoking (ref: current smoker) | | | | |
| Ex-smoker | -0.16 | 0.02 | -0.30 to -0.023 |  |
| Never smoked | -0.12 | 0.09 | -0.25 to 0.019 |  |
| Paid work | -0.09 | 0.1 | -0.20 to 0.027 |  |
| IMD | -0.057 | <0.0001 | -0.075 to -0.039 |  |
| Comorbidity | 0.051 | 0.04 | 0.036 to 0.099 |  |
| Seropositive | -0.14 | 0.008 | -0.24 to -0.035 |  |
| Symptom duration (ref: <1 month) | | | |  |
| 1 to 6 months | -0.03 | 0.7 | -0.21 to 0.14 |  |
| >6 months | 0.10 | 0.3 | -0.088 to 0.28 |  |
| Prompt referral | -0.032 | 0.5 | -0.13 to 0.069 |  |
| Prompt therapy commencement | -0.14 | 0.008 | -0.25 to -0.036 |  |
| Initial DMARD regimen (ref: no DMARD) | | | |  |
| Monotherapy | -0.18 | 0.1 | -0.40 to 0.050 |  |
| Combination therapy | 0.22 | 0.4 | -0.28 to 0.73 |  |
| Baseline Corticosteroids | 0.24 | 0.02 | 0.044 to 0.45 |  |
| DMARD monotherapy + corticosteroids | 0.052 | 0.7 | -0.20 to 0.30 |  |
| DMARD combination therapy + corticosteroids | -0.39 | 0.2 | -0.92 to 0.15 |  |

Table 9. Predictors of 12 months DAS28.

| 12 months DAS28 | Beta-coefficient | p value | 95% Confidence interval |  |
| --- | --- | --- | --- | --- |
| Age | -0.011 | 0.002 | -0.019 to -0.0042 |  |
| Female gender | 0.24 | 0.008 | 0.065 to 0.42 |  |
| BAME | -0.030 | 0.8 | -0.29 to 0.23 |  |
| Smoking (ref: current smoker) | | | | |
| Ex-smoker | -0.23 | 0.07 | -0.47 to 0.015 |  |
| Never smoked | -0.17 | 0.2 | -0.40 to 0.070 |  |
| Paid work | -0.20 | 0.04 | -0.39 to -0.052 |  |
| IMD | -0.047 | 0.002 | -0.077 to -0.017 |  |
| Comorbidity | 0.130 | 0.002 | 0.050 to 0.21 |  |
| Seropositive | -0.090 | 0.3 | -0.27 to -0.086 |  |
| Symptom duration (ref: <1 month) | | | |  |
| 1 to 6 months | -0.19 | 0.2 | -0.48 to 0.11 |  |
| >6 months | 0.53 | 0.7 | -0.026 to 0.37 |  |
| Prompt referral | -0.071 | 0.4 | -0.25 to 0.10 |  |
| Prompt therapy commencement | -0.081 | 0.4 | -0.26 to -0.094 |  |
| Initial DMARD regimen (ref: no DMARD) | | | |  |
| Monotherapy | -0.25 | 0.2 | -0.65 to 0.15 |  |
| Combination therapy | -0.36 | 0.4 | -1.21 to 0.49 |  |
| Baseline Corticosteroids | 0.18 | 0.3 | -0.18 to 0.53 |  |
| DMARD monotherapy + corticosteroids | 0.086 | 0.7 | -0.36 to 0.53 |  |
| DMARD combination therapy + corticosteroids | 0.17 | 0.7 | -0.72 to 1.06 |  |

**References**

1. Rubin DB. Multiple imputation for survey nonresponse. New York: Wiley; 1987.
